# Estradiol mediates colonic epithelial protection in aged mice after stroke and is associated with shifts in the gut microbiome

**DOI:** 10.1080/19490976.2023.2271629

**Published:** 2023-11-01

**Authors:** Juneyoung Lee, Pedram Peesh, Victoria Quaicoe, Chunfeng Tan, Anik Banerjee, Patrick Mooz, Bhanu P. Ganesh, Joseph Petrosino, Robert M. Bryan, Louise D. McCullough, Venugopal Reddy Venna

**Affiliations:** a Department of Neurology, McGovern Medical School, The University of Texas Health Science Center at Houston, Houston, TX, USA; b Alkek Center for Metagenomics and Microbiome Research, Department of Molecular Virology and Microbiology, Baylor College of Medicine, Houston, TX, USA; c Department of Anesthesiology, Baylor College of Medicine, Houston, TX, USA; d Department of Neurology, Memorial Hermann Hospital-Texas Medical Center, Houston, TX, USA

**Keywords:** Ischemic stroke, gut epithelium, gut-brain axis, sex differences, aging

## Abstract

The gut is a major source of bacteria and antigens that contribute to neuroinflammation after brain injury. Colonic epithelial cells (ECs) are responsible for secreting major cellular components of the innate defense system, including antimicrobial proteins (AMP) and mucins. These cells serve as a critical regulator of gut barrier function and maintain host-microbe homeostasis. In this study, we determined post-stroke host defense responses at the colonic epithelial surface in mice. We then tested if the enhancement of these epithelial protective mechanisms is beneficial in young and aged mice after stroke. AMPs were significantly increased in the colonic ECs of young males, but not in young females after experimental stroke. In contrast, mucin-related genes were enhanced in young females and contributed to mucus formation that maintains the distance between the host and gut bacteria. Bacterial community profiling was done using universal amplification of 16S rRNA gene sequences. The sex-specific colonic epithelial defense responses after stroke in young females were reversed with ovariectomy and led to a shift from a predominately mucin response to the enhanced AMP expression seen in males after stroke. Estradiol (E_2_) replacement prior to stroke in aged females increased mucin gene expression in the colonic ECs. Interestingly, we found that E_2_ treatment reduced stroke-associated neuronal hyperactivity in the insular cortex, a brain region that interacts with visceral organs such as the gut, in parallel to an increase in the composition of *Lactobacillus* and *Bifidobacterium* in the gut microbiota. This is the first study demonstrating sex differences in host defense mechanisms in the gut after brain injury.

## Introduction

Ischemic stroke is a leading cause of mortality and leads to persistent neurological disability that is seen in many stroke survivors.^1^ Importantly, epidemiological studies suggest that stroke is a sexually dimorphic disease, with differences in incidence between males and females across the lifespan.^2,3^ The significant contribution of sex hormones to host immune responses in infectious and autoimmune diseases has been well documented.^4–6^ The role of sex hormones (e.g., estrogen) has been extensively studied in rodent stroke models. Numerous studies have shown that estrogen is neuroprotective by reducing infarct size, neuronal damage and hypoperfusion,^7–12^ but some studies have reported a deleterious role of estrogen,^13–15^ including in aged mice.^16,17^ In humans, a protective role of estrogen on stroke had been reported,^18–21^ however several randomized double-blinded studies, including the large Women’s Health Initiative (WHI),^22,23^ found that hormone therapy (HT) can increase the risk of stroke without a reduction of mortality, especially in women over 65 y.^24–26^ Further sub-analysis of the WHI results by age group, and more recent randomized control studies, including the Kronos Early Estrogen and Prevention Study (KEEPs) and Early versus Late Intervention Trial with Estradiol (ELITE),^27^ demonstrate that the risk of adverse cardiovascular events for HT is low for women <60 y of age or within 10 y from menopause, showing the importance of the timing of initiation of hormone therapy on vascular risk. More recent investigations have revealed that both estrogens and gut microbiota can affect outcomes after stroke differently in males and females.^28,29^

It has become apparent that the state of the microbiome and the physiological health of the gut plays an important role in stroke outcome. A large body of evidence has identified a bidirectional system of communication between the gut and the brain after stroke.^30–33^ Through “top-down” signaling of the brain-gut axis, stroke disrupts the normal function of the gut and alters the gut microbiota.^31,32^ Stroke leads to gut dysbiosis, defined as a pathological shift in the microbiota, and increased gut permeability, leading to an exacerbation of neuroinflammation.^31,32,34,35^ However, there are limited studies investigating if estrogen-mediated mechanisms are involved in the gut response to stroke. Whether estrogen contributes to gut barrier integrity and leads to a reduction in invading antigens and bacteria following stroke, is unknown.

In order to maintain intestinal homeostasis, intestinal epithelial cells (ECs) must respond to environmental signals. ECs have evolved protective mechanisms to maintain host barrier function to keep the microbiota at bay; by producing mucin (Muc) and antimicrobial proteins (AMPs).^36,37^ Mucins are secreted by goblet cells, a specialized type of ECs, and form a thick mucus layer that serves as a protective interface separating the luminal microbiota from the ECs.^38^ AMPs, including regenerating islet-derived protein 3 genes (Reg3), are produced by specialized ECs and fortify the mucosal protective barrier through their antimicrobial effects.^36,37^

In this study, we hypothesized that stroke differentially alters the epithelial responses and the protective barrier function in a sexually dimorphic manner. We narrowed our focus to the colon since it harbors the greatest load and diversity of microbes.^39^ As endogenous gonadal hormones are known to contribute to sex differences, and estrogens are potent neuroprotectants following experimental ischemic stroke,^40^ we performed ovariectomy (OVX) in young females to examine the potential link between estrogens and EC-regulated gut protective mechanisms. Lastly, exogenous 17β-estradiol (E_2_) was given to aged female mice to determine if the female-specific protective mechanisms could be recapitulated in aged animals. We show that estrogen and the host microbiome play a coordinated role in stroke-induced colonic epithelial damage through mucin and AMP-regulated inflammatory responses that differ by sex.

## Results

### Stroke increases Reg3 family-specific antimicrobial genes in young male colonic ECs

**Figure 1.**
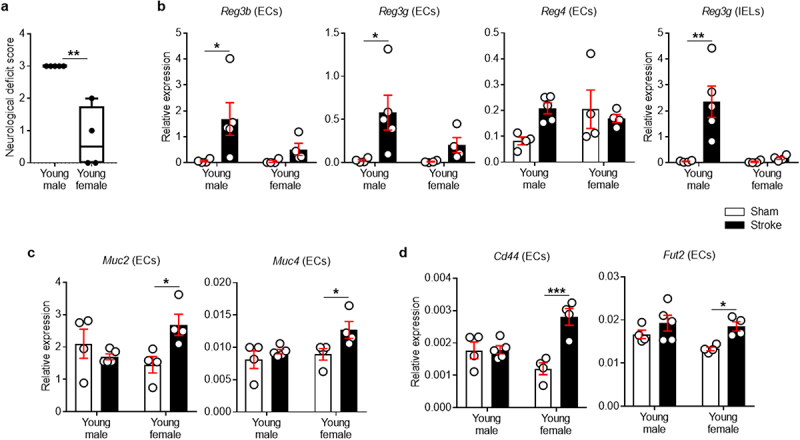
Stroke increases Reg (males) and mucin-related genes (females) in colonic ECs.

We first examined the neurological deficit scores (NDS) of young males and females after stroke. One female mouse was excluded from the study as they died during the post-operative care period. As shown in Figure 1a, young females had lower NDS at post-MCAO day 7 compared to young males. In order to investigate potential sex differences in the epithelial antimicrobial responses in the colon after stroke, we isolated ECs and intraepithelial lymphocytes (IELs) from colonic tissue following a 60-minute middle cerebral artery occlusion (MCAO) and examined the expression of AMPs. As ECs and IELs both express genes for AMP proteins, e.g., Reg3γ,^41,42^ we separated ECs from IELs using Percoll density gradients, as in recent reports.^43,44^ We found that young male mice increased the mRNA expression of multiple Reg3 genes at day 7 post-stroke compared to young sham mice (Figure 1b). *Reg3b*, *Reg3g* and *Reg4* increased ~24-fold (*P* = .0215), ~21-fold (*P* = .0161) and ~3-fold (*P* = .0659) on average, respectively. However, young female mice had no significant change in these AMP genes after stroke. We also evaluated the expression of other subtypes of AMPs that contribute to the protection of the epithelial barrier in the gut. Notably, stroke did not induce changes in *Defa* (for α-defensin) and *Lyz1* (for lysozyme) genes in any of the groups (data not shown). These results indicate that AMP expression in young males is Reg3-specific after stroke.

(a) At day 7 after a 60-minute MCAO, neurological deficit scores (NDS) were assessed. (b) Young male-specific increases in Reg3 family genes (*Reg3b* and *3 g*) in isolated colonic ECs following MCAO. (c) Young female-specific increase in mucin genes (*Muc2* and *Muc4*) in colonic ECs at post-MCAO day 7. (d) Young female-specific increase in *Cd44* and *Fut2* genes in colonic ECs at post-MCAO day 7. Beta-actin was used to normalize mRNA levels of ECs. Data shown are mean±SEM. Two-way ANOVA method was used to analyze data with two factors including sex and control/stroke, followed by the post-hoc stroke vs control group comparison within male and female, respectively. Multiple testing adjustment was performed by Sidak method. For NDS data, Mann–Whitney *U* test of unpaired *t* test was used. *n* = 4–5 per group. *, *P*<.05, **, *P*<.01, ***, *P*<.001.

### Young male lymphocytes within the colonic epithelium had increased expression of proinflammatory genes after stroke

IELs are a lymphocyte population intercalated between ECs in the intestinal epithelium. Both ECs and IELs play an essential role in host-microbiota homeostasis in the gut. In injured epithelia, IELs control the paracellular penetration of commensal bacteria by inducing both antibacterial and pro-inflammatory responses.^45^ Thus, we asked if the closer contact of colonic epithelial surface with luminal bacteria in young males provokes immune responses from IELs after stroke. We isolated IELs from the same mice which were used for the EC study and quantified the expression of genes including *Reg3g*, *Cxcl9*, *Il1b* and *Cxcl2*/*Mip2a*. These have been previously recognized as microbiota-associated factors that are produced by IELs following epithelial damage.^45,46^ As shown in Figure 1b, only young male mice increased antimicrobial *Reg3g* expression after stroke (*P* = .0011). Further, young male mice had significantly higher expression of pro-inflammatory and chemotactic cytokines including *Cxcl2*/*Mip2a* (*P* = .0171), but not *Cxcl9* and *Il1b*, after stroke, which was not seen in young females (Supplementary Figure S1). Given that IELs are situated on the basolateral side of ECs near tight junctions, our data supports the idea that the antigens approach and penetrate the host epithelium after stroke, resulting in the activation of IELs in response to the invading bacteria in young males after stroke.

### Stroke enhances mucin-related genes in young female colonic ECs

Mucins provide the gut with a densely packed physical barrier that serves as the primary line of defense in mucosal protection.^47^ Young male mice had no increase in *Muc2* or *Muc4* genes in their colonic ECs. However, in young female mice, there was a significant increase in mucin genes after stroke (Figure 1c, *P* = .0234 for *Muc2* and *P* = .0434 for *Muc4*). CD44 signaling is critical for promoting both EC proliferation^48^ and mucus secretion,^49^ and therefore, we examined CD44 gene expression in colonic ECs. Stroke induced an increase in colonic epithelial *Cd44* expression only in young female mice (*P* = .0003; Figure 1d). To further elucidate the stroke-induced changes specific to young female mice, epithelial glycosylation was examined. The expression level of *Fut2*, a key enzyme that regulates the process of fucosylation,^50^ was significantly increased in colonic ECs from young female mice (*P* = .0330; Figure 1d). In contrast, there was no significant change in *Fut2* mRNA levels in young males after stroke.

### Young females maintain larger spatial separation between the colonic epithelium and commensal bacteria after stroke compared with young males

**Figure 2.**
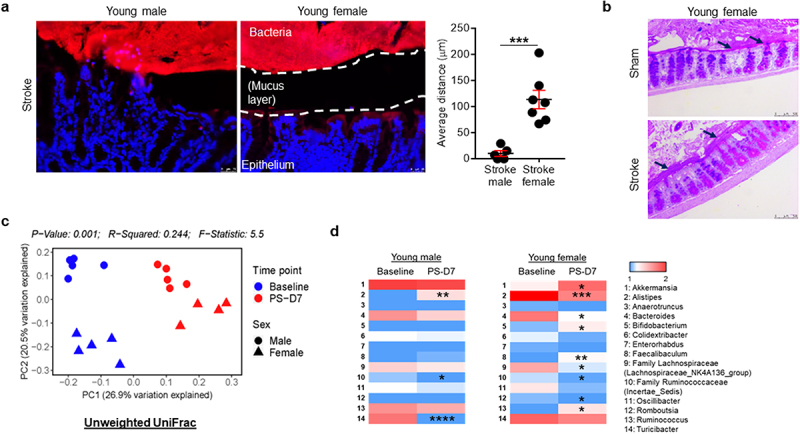
Spatial segregation between the host colonic epithelium and commensal microbiota and fecal microbiota profiles differ in young male and female mice.

As mucin gene expression was significantly increased in young female mice after stroke, we hypothesized that the mucus layer would be thicker in the colonic region of young females, providing an increased barrier between the host and the luminal bacteria in the colon. For direct and comprehensive visualization of the entire bacterial population in the colon, bacterial fluorescence in situ hybridization (FISH) was performed by a blinded investigator, using a universal probe EUB338, which is specific for the bacterial 16S rRNA gene.^51^ After stroke, young male mice failed to maintain this distance, and had more disruption of colonic crypt structures (Figure 2a). The colonic ECs in male mice were in close contact with bacteria, while in young females the crypt epithelial architecture remained intact and the distance between host and bacteria was maintained (Figure 2a). In addition, we performed Periodic acid – Schiff (PAS) staining to assess the number of goblet cells and their mucin production within the intestinal epithelium. Figure 2b demonstrates that young female mice had a thicker mucus layer compared to sham counterparts, suggesting that goblet cell function was not affected after stroke in young females, further supporting our bacteria FISH data shown in Figure 2a. The number of goblet cells remained the same across the experimental groups (data not shown).

(a) The spatial segregation between colonic epithelium and bacteria in young mice at pre-stroke and post-MCAO day 7. Fixed tissue sections were hybridized with Cy3-labeled EUB338 probe (red) and counterstained with DAPI (blue). The closest proximity of crypts to bacteria in stroke mice (three crypts per each section) was measured using ImageJ by an investigator blinded to group. Data shown are mean±SEM. Student’s *t*-test was used after the normality of data was confirmed by the Shapiro–Wilk normality test. *n* = 5–7 per group. ***, *P*<.001. (b) Representative images show the number of goblet cells and mucus layer thickness assessed by PAS staining on colonic tissues from young female mice after stroke. (c) Beta-diversity of fecal microbiota in young male and female mice at baseline and PS-D7. The data was analyzed by ATIMA and plotted by PCoA using unweighted UniFrac distance. *n* = 4–5 per group. (d) Raw reads of 14 identified genus are log 10-transformed to generate intensity heatmaps, each row graphically represents the median difference at post-stroke day 7 compared to their respective baseline group. *n* = 4–5 per group. *P<.05, **P<.01, ***P<.001, ****P<.0001 by unadjusted Mann–Whitney *U* test.

### Stroke induces shifts in the gut microbiota composition in both young females and males

We employed 16S rRNA sequencing (16S rRNA-seq) using fecal samples to assess the gut microbiota composition and diversity in young mice of both sexes, before and after stroke (post-stroke day 7; PS-D7). Importantly, upon visualization of beta-diversity or between-samples diversity, with unweighted UniFrac distance by principal coordinate analysis (PCoA), we observed a notable clustering effect between the groups (Figure 2c). To further explore specific microbiota differences, we compared the 16S rRNA-seq data and analyses of relative abundances were limited to taxa classified at the genus level in the metagenomics datasets. When analyzing the data with a criterion of taxa with a *P* < .2 in either male or female groups at the genus level, we identified the 14 bacterial taxa using a Mann–Whitney *U* test (Figure 2d). We found that *Alistipes* was increased; whereas *Turicibacter* and Family *Ruminococcaceae* (*Incertae_Sedis*) were reduced in young males after stroke (Figure 2d). In contrast, young female mice showed changes of different genera including *Akkermansia*, *Alistipes*, *Bacteroides*, *Bifidobacterium*, *Faecalibaculum*, Family *Lachnospiraceae* (*Lachnospiraceae*_NK4A136_group), Family *Ruminococcaceae* (*Incertae_Sedis*), *Romboutsia* and *Ruminococcus* after stroke (unadjusted *P* < .05; Figure 2d). Taken together, these results indicate that the gut microbiome dynamically responds to stroke-induced gut dysfunction in a sex-specific manner.

### Ovariectomy worsens neurological deficits and alters epithelial defense mechanisms of young female mice after stroke

**Figure 3.**
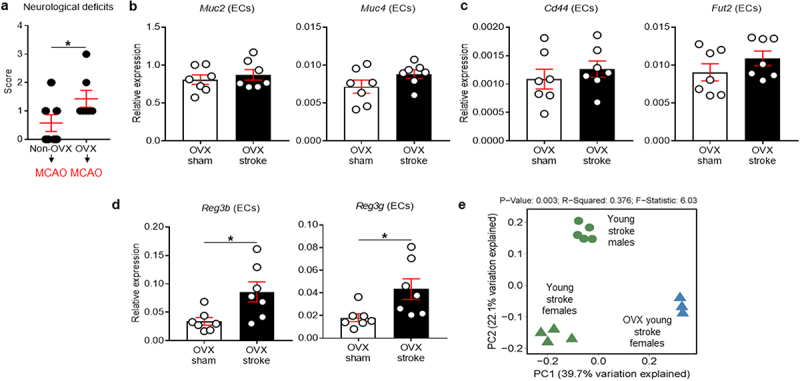
Ovariectomy worsened neurological deficits and switches the protective mechanisms in the colonic epithelium of young females to a male-like pattern.

Estrogens, primarily estradiol (E_2_), have a protective role in experimental stroke,^52,53^ and this neuroprotective effect can be abolished by OVX and restored with exogenous E_2_ replacement.^54^ Therefore, to understand the effect of ovarian hormones on the colonic epithelial responses to stroke, and to determine whether the mucin response is mediated by hormones, we ovariectomized young female mice 14 d prior to stroke and isolated colonic ECs at post-stroke day 7. Two female mice with both OVX and MCAO surgeries were excluded from the study as they died during the post-operative care period. We first examined if the removal of gonadal hormones affects post-stroke neurological deficits in young female mice. We found that OVX females had worse neurological deficits at post-stroke day 7, compared with non-OVX females (*P* = .0449; Figure 3a). More interestingly, OVX led to the loss of the stroke-induced increase in mucin-related genes (Figure 3b,c) that was seen in intact females after stroke. Instead, ovariectomized female mice had an up-regulation of AMP genes such as *Reg3b* and *Reg3g* (*P* = .0177 for *Reg3b* and *P* = .0228 for *Reg3g*; Figure 3d), similar to the post-stroke response seen in young males. This implies that the lack of gonadal hormones (hormone-driven) or OVX-induced microbiota alteration (microbiota-driven) may cause female mice to initiate an epithelial response similar to that observed in males. To address these two possibilities and to determine if these changes were mediated specifically by hormonal effects on the epithelium in young females, we performed 16S rRNA-seq using fecal samples from OVX stroke mice and compared with the microbiome of non-OVX stroke mice. Interestingly, the microbiome between young males, young females and OVX females was distinctly clustered in PCoA analysis at day 7 after MCAO (Figure 3e). These data suggest that female gonadal hormones are a critical factor driving colonic mucin-related gene expression after stroke in young female mice and that sex differences in the protective strategies mounted by males (AMPs) and females (mucins) are hormonally controlled in young mice. The changes in gene expression in post-MCAO colonic ECs in both young males and females (Figure 1b–d) and in OVX females (Figure 3b–d) were further compared (see Supplementary Figure S2). When analyzed our 16S rRNA-seq data to detect similarities at the genus level in young males and young OVX females compared to young ovary-intact females after stroke, the relative abundance of *Alistipes*, *Bifidobacterium*, *Dubosiella* and *Turicibacter* was different between young males and young ovary-intact females. In addition, the relative abundance of *Akkermansia*, *Alistipes*, *Bacteroides*, *Bifidobacterium*, *Colidextribacter*, *Faecalibaculum*, *Monoglobus*, *Oscillibacter*, *Roseburia* and *Turicibacter* was different between OVX females and ovary-intact females (Supplementary Figure S3), further supporting that the microbiota changes could be involved in the gut response to ischemic brain injury.

(a) MCAO was performed 2 weeks after OVX in young female mice. At post-MCAO day 7, NDS was assessed and compared with non-OVX stroke females. Colonic ECs were isolated from sham or stroke OVX mice and mRNA expression of *Muc2* and *4* (b), *Cd44*, *Fut2* (c), *Reg3b* and -*3 g* (d) was examined using RT-qPCR. Beta-actin was used to normalize mRNA levels. Data shown are mean±SEM. Mann–Whitney *U* test (a) or Student’s *t*-test (b to d) were used after the normality of data was confirmed by the Shapiro–Wilk normality test. *n* = 7 per group. *, *P*<.05. (e) 16S rRNA-seq data for beta-diversity or between-community diversity at post-MCAO day 7 with Bray-Curtis distances plotted by PCoA using ATIMA analysis.

### E_2_ replacement enhances mucin gene expression in the colonic epithelium of aged females after stroke

**Figure 4.**
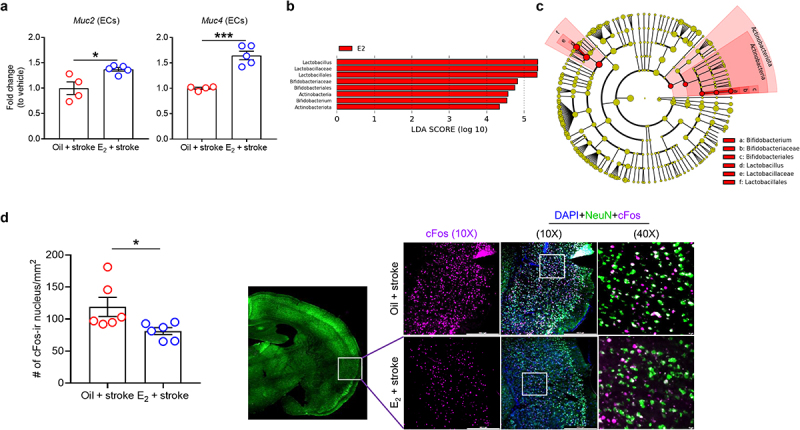
Replacement of E_2_ restores mucin gene expression in colonic ECs, alters microbiome profiles, and reduces a marker of neuronal hyperactivity in the brain of aged stroke females.

Finally, to determine if the female-specific colonic defense mechanisms were also seen in aged animals, and if these were mediated by estrogen, we examined gut epithelial mucin expression in reproductively senescent female mice. Aged females (18–20 months), which have low E_2_ levels compared to young and middle-aged female mice,^55^ were supplemented with E_2_^16^ for 2 weeks. The mice were subjected to MCAO and were euthanized at day 7. Five aged female mice (two MCAO mice with oil treatment and three MCAO mice with E_2_ treatment) were excluded from the study as they died during the post-operative care period. Next, we isolated colonic ECs from vehicle- and E_2_-treated aged stroke females and examined the expression levels of mucin genes. Interestingly, E_2_-replaced stroke mice showed higher expression of *Muc2* (*P* = .0156) and *Muc4* (*P* = .0003), compared with the vehicle-treated group (Figure 4a). Taken together, our data supports the idea that estrogen exerts beneficial effects on the gut after stroke.

MCAO was performed 2 weeks after treating females with E_2_ or vehicle (sesame oil) in aged female mice (18–20 months). At post-MCAO day 7, colonic ECs were isolated from mice and the mRNA expressions of *Muc2* and *4* (a) were examined using RT-qPCR. Beta-actin was used to normalize mRNA levels. Data shown are mean±SEM. Student’s *t*-test was used after the normality of data was confirmed by the Shapiro–Wilk normality test. *n* = 4–5 per group. *, *P*<.05, ***, *P*<.001. (b) Discriminant taxa between aged stroke females treated with oil (vehicle) and E_2_ were ranked by their linear discriminant analysis (LDA) scores (*n* = 5 per group). (c) Cladogram using LEfSe analyzed the hierarchical organization reflecting bacterial taxonomies. (d) Quantification and representative immunohistochemical images for cFos staining in the insular cortex in oil-treated or E_2_-treated aged stroke female mice. Mann–Whitney *U* test was used. *n* = 6 per group. *P<.05.

### E_2_ replacement alters the gut microbiota composition after stroke and reduces a marker of neuronal activity in the insular cortex in aged stroke females

To further investigate whether the gut microbiota composition is associated with E_2_ levels in aged females after stroke, we used linear discriminant analysis (LDA) effect size to identify bacterial taxa that were differently enriched in fecal samples between mice treated with oil (vehicle) and E_2_ prior to stroke (*P* < .05, LDA score (log10) > 4.0). Across the two groups, a total of 8 enriched bacterial taxa were identified (Figure 4b). In the E_2_-treated mice, *Lactobacilus*, *Lactobacillaceae*, *Lactobacillales*, *Bifidobacteriaceae*, *Bifidobacteriales*, *Actinobacteria*, *Bifidobacterium* and *Actinobacteriota* were higher compared to the oil-treated group. All bacterial taxa identified across the two groups are listed from the highest to the lowest LDA scores and filtered based on the significance level (*P* < .05). Figure 4c represents the LEfSe results as a cladogram. When analyzed to identify any similarities in microbiota compositions of both young intact and E_2_-treated aged stroke female mice, we found a trend toward an increase in the relative abundance of *Bifidobacterium* in both groups when compared to their control groups (Supplementary Figure S4). Of note, *Bifidobacterium* species are well-known mucus enhancers in the colon by secreting beneficial metabolites such as short-chain fatty acids and neurotransmitters.^56,57^

It is known that estrogen can regulate neuronal activity in the insular cortex after stroke.^58^ In addition, this region is highly associated with gut inflammation.^59^ Therefore, we measured cFos in the insular cortex.^60^ We found that aged female mice with E_2_ supplementation had significantly reduced cFos-positive neurons compared to the oil (vehicle)-treated group (*P* < .05; Figure 4d), suggesting a dampening of an aberrant neuronal activation in the post-stroke brain.

## Discussion

The intestinal epithelium is a critical interface for host-microbe symbiosis as it mediates the crosstalk between microbes and immune cells. We found that cells in the colonic epithelium (ECs and IELs) actively participated in stroke-induced and microbe-associated gut alterations. Estrogen reduces brain injury in experimental stroke via neuroprotective and anti-inflammatory actions.^40,61^ In this study, we extend the beneficial effects of estrogen to the gut. Our data shows that female gonadal hormones drive gut defense mechanisms in young female mice after stroke. Further, we demonstrated that loss of gonadal hormones altered gut defense mechanisms in female mice after stroke. Removal of the ovaries led to a loss of the stroke-induced increase of mucin genes, and instead, led to a significant increase in AMP genes, similar to males. Most importantly, this appears to be mediated by E_2_, as estrogen supplementation in aged female mice prior to stroke led to enhanced expression of Muc2 and Muc4 in the gut epithelium, similar to that seen in ovary-intact females.

It is well documented that young female mice are protected after experimental stroke compared to young and aged male mice and aged females.^62^ In the gut, we found that ischemic stroke provoked a colonic response, and that these responses were sex-specific. Young male mice showed enhanced Reg-specific AMPs, e.g., *Reg3b* and *Reg3g*, while young females showed increased mucin-related gene expressions, e.g., *Muc2* and *Muc4*. In addition, we profiled the bacterial compositions of the gut in young mice of both sexes after stroke to identify a possible role of the microbiome in stroke-initiated brain to gut signaling. Our data showed that stroke induces highly unique alterations in the fecal microbiota of mice in a sex-dependent manner. Young female mice preserve their colonic epithelial barrier by enhancing mucins after stroke. This host-bacteria interaction may be secondary to E_2_-enhanced mucin production in the colonic epithelium after stroke.

Previous studies have shown that the decline in estrogen levels after menopause reduces *Lactobacilli* dominance in the vaginal microenvironment, and estrogen replacement therapy restores this dominance.^63,64^ Here, we found that E_2_ supplementation increases the fecal composition of *Lactobacillus* and *Bifidobacterium* in aged stroke mice. Interestingly, these gut microbes produce β-glucosidase enzymes that can convert inactive estrogens into their active forms through deconjugation.^65,66^ Estrogens regulate immune and epithelial cell responses by regulating the release of bacterial metabolites to dampen inflammation, and by blocking the activation of endotoxins such as lipopolysaccharides. Taken together, our findings suggest that estrogens can reshape the gut microbiome and protect female-specific colonic epithelial cell responses to stroke.

The insular cortex regulates the connectivity between afferent and efferent fibers of visceral organs.^67^ Increasing evidence suggests that the insula regulates the inflammatory status of the gut.^59^ Previous studies have shown that estrogen reduces neuronal excitability and stroke-induced cell death in the insular cortex; however, these studies only examined changes at acute time points and only assessed young animals, and gut responses were not evaluated.^58^ Here, we found that E_2_ supplementation in aged female mice prior to stroke has the potential to reduce the levels of cFos in the ipsilateral insula, suggesting that reduced neuronal excitability might be associated with the alterations in colonic EC responses regulated by E_2_ after stroke.

It is well known that estrogen is neuroprotective;^40^ one important caveat of this study is that some of the colonic changes seen may be related to the size of the infarct. In addition, post-stroke outcomes and functional recovery are also mediated by the sex chromosome compliment (XX vs. XY).^68^ However, sex-specific epithelial responses in the gut have not been previously investigated, adding considerable novelty to our findings. Thus, future investigations of how infarct size and location contribute to the colonic epithelial responses after stroke and examination of the changes in EC responses at chronic time points after stroke are needed. Future studies examining post-stroke colonic epithelial responses that incorporate a mouse model such as the four-core genotype model (i.e., XX and XY males and XX and XY females) to differentiate the role of gonadal hormones vs. the sex chromosome complement are also warranted. Other limitations of the study include 1) Changes were analyzed at only one timepoint (post-stroke day 7); 2) The lack of deep microbiome profiling, using shotgun sequencing; 3) The neuronal activity was analyzed using one marker (cFos); and 4) The possibility of involvement of other brain areas and detailed electrophysiological studies are still needed.

In conclusion, our current study demonstrates that the colonic epithelium, which contributes to both mechanical and immunologic responses that regulate host-microbiota crosstalk in the gut, dynamically responds to brain injury. Importantly, several key chemical and biological niches in the colonic epithelium that protect the host against harmful factors initiated by brain injury, including mucins and AMPs were significantly altered in mice after stroke. These changes in the host colon, and in the microbiota composition, were sex-specific and were estrogen dependent. Our findings demonstrate the importance of studying both sexes and further highlight potential interactions between sex, the gut microbiome and immunity in age-related neurological diseases such as stroke.

## Materials and methods

### Mice

All animal protocols were approved by the Institutional Animal Care and Use Committee at the University of Texas Health Science Center at Houston (UTHealth). This study was performed in accordance with the guidelines provided by the National Institutes of Health (NIH) and followed RIGOR guidelines.^69^ Young male and female C57BL/6J mice (2-month) were purchased from Jackson Laboratory and acclimatized for a minimum of 1 month before use. Aged female C57BL/6J mice (18–20 months) were raised in our animal facilities. All mice were housed in ventilated cages with irradiated corncob bedding (1/8”), mice were received filtered tap water and irradiated and nutritionally balanced rodent diet (PicoLab® rodent diet 20 5053, LabDiet). To determine the effect of female gonadal hormones, one cohort of young female mice was ovariectomized 2 weeks prior to stroke. Another cohort of aged female mice was subcutaneously implanted with E_2_ pellets (Sigma-Aldrich) or sesame oil as a vehicle 2 weeks prior to stroke.^70^

### Experimental stroke

A 60-minute middle cerebral artery occlusion (MCAO) was achieved by inserting a silicone coated suture (0.21 or 0.23 mm diameter for young and aged mice respectively) into the external carotid artery (ECA).^33,71^ The suture was advanced into the circle of Willis of mice to occlude the MCA. The animal was awakened from anesthesia and intra-ischemic focal deficits were confirmed. After 60 minutes, the mouse was re-anesthetized and the suture was removed allowing for reperfusion. Sham control mice underwent the same surgical procedure and exposure of the ECA but the suture was not inserted into the MCA. All mice were randomly assigned to stroke or sham cohorts. All surgeries were conducted using isoflurane anesthesia with temperature maintenance.

### Isolation of colonic ECs and intraepithelial lymphocytes (IELs)

Colonic ECs and IELs were isolated at post-stroke day 7, as previously described.^43,44^ Briefly, colonic tissue was harvested from sham and stroke mice at day 7 and analyzed by an investigator blinded to treatment group. After removal of the cecum, the tissues were opened longitudinally and rinsed with ice-cold RPMI-1640 (Corning). The tissue was incubated with RPMI-1640 containing 2 mM EDTA (Invitrogen) and 10% FBS (Gibco) for 30 min at 37°C. The resulting solution containing digested tissues was passed through a nylon mesh (70 μm, Falcon). The filtrate was applied to Percoll (GE Healthcare) density gradients: 25%, 40% and 75%. After centrifugation at 500 g for 20 min, the interface between gradients were collected to obtain ECs (25% and 40%) and IELs (40% and 75%).

### Quantification of mRNAs

Total RNA was isolated from the colonic ECs and IELs using Trizol (Invitrogen). cDNA was synthesized from 500 ng of RNA using iScript Reverse Transcription Supermix for RT-qPCR kit (Bio-Rad). Beta-actin and glyceraldehyde-3-phosphate dehydrogenase (GAPDH) were used as internal controls to normalize the expression level of ECs and IELs mRNAs, respectively. Real-time quantitative PCR (RT-qPCR) using cDNA was performed by using SsoAdvanced^TM^ Universal SYBR^Ⓡ^ Green Supermix (Bio-Rad) in CFX96 Touch^TM^ Real-Time PCR Detection System (Bio-Rad). The list of primers used in this study along with their sequences are listed in Supplementary Table S1.

### Bacterial fluorescence in situ hybridization (FISH) and PAS staining

Bacterial FISH was performed as previously described.^72^ Briefly, colonic tissue was isolated from sham and stroke mice at day 7 and fixed in Carnoy’s solution. Paraffin-embedded tissues were sectioned at 5 μm. For bacterial FISH, the sections were hybridized with Cy3-labeled EUB338 probe (primer sequence: GCTGCCTCCCGTAGGAG) at 51°C overnight (Integrated DNA Technologies). The hybridized sections were co-stained with 4’,6-diamidino-2-phenylindole (DAPI, Sigma-Aldrich). The slides were analyzed by confocal microscopy at a wavelength of 330 to 700 nm (Leica DMi8 and TCS SPE microsystems) by a blinded investigator. For PAS staining, colonic tissue sections were subjected to periodic acid (0.5% solution) for 5 min and Schiff reagent (basic fuchsin, sodium metabisulfite and 1N hydrochloric acid in distilled water) for 15 min. The slides were analyzed by bright field microscopy by a blinded investigator.

### 16S ribosomal RNA sequencing (16S rRNA-seq) and analysis

Fecal samples (i.e., stool samples) were collected from sham and stroke mice between 9 and 10 am at baseline and at post-stroke day 7 and immediately frozen at −80°C until the analysis. Fecal DNA was extracted using MO BIO’s PowerMag Soil DNA Isolation kit (MO BIO). Using high-throughput sequencing (Illumina MiSeq platform; Illumina, San Diego, CA), bacterial taxa from the fecal samples were analyzed via amplification of the V4 region of the 16S rRNA gene.^32,73^ The data was analyzed using the Agile Toolkit for Incisive Microbial Analyses (ATIMA), a software developed by the Alkek Center for Metagenomics and Microbiome Research (CMMR) at the Baylor College of Medicine.^74^ Galaxy online interface Version 1.0 (https://huttenhower.sph.harvard.edu/galaxy/) was used to perform linear discriminant analysis (LDA) and to generate cladogram and LDA effect size (LEfSe) plots.^75^

### Immunohistochemistry

Brain tissues were collected from perfused mice and sectioned at a 30-micron thickness. Free floating brain sections were incubated in blocking solution followed by antigen retrieval (BioGenex, HK086-9K) as per manufacturer’s instructions. Sections were incubated in blocking solution (2% donkey normal serum + 0.1% BSA + 0.2% Triton X-100 in PBS) for 1 h at room temperature, followed by the incubation with anti-cFos (Oncogene Research Products, PC38; 1:20,000); Alexa Fluor 488 pre-conjugated anti-NeuN (EMD Millipore, MAB377X; 1:500) in blocking solution overnight at room temperature. The sections were washed with PBS three times for 5 min, and then Alexa Fluor 647 pre-conjugated donkey anti-rabbit IgG (H+L) (Jackson ImmunoResearch Laboratories, 711-605-152; 1:2,000) was used as secondary antibody to detect the cFos expression. The sections were mounted on slides and then stained with DAPI (Sigma-Aldrich, F6057). Sections were imaged using Leica Thunder Imager DMi8 microscope (Leica, Heerbrugg, Switzerland). Cell counts from images were analyzed using ImageJ by an investigator blinded to treatment condition.

### Statistics

Two-way ANOVA was used to analyze data with two factors including sex and control/stroke, followed by the post-hoc stroke vs control group comparison within males and females, respectively. Multiple testing adjustment was performed by Sidak method. For two group comparisons, Student’s *t*-test or Mann–Whitney *U* test were used and the normality of data was confirmed by the Shapiro–Wilk normality test. *P* value less than 0.05 was considered as significant. GraphPad Prism 7.03 was used for all data analysis.

## Supplementary Material

Supplemental Material

## Data Availability

The raw reads of Illumina sequencing have been submitted to the Sequence Read Archive (SRA) in the National Center for Biotechnology Information (NCBI) under BioProject accession number PRJNA628128 https://www.ncbi.nlm.nih.gov/bioproject/PRJNA628128. All data sets and supporting material details of this study are available from the corresponding or first author of the article upon reasonable request from qualified investigators.
