# Supplementary material for: Estradiol mediates colonic epithelial protection in aged mice after stroke and is associated with shifts in the gut microbiome
Source: Gut Microbes. 2023 Nov 1;15(2):2271629. doi: 10.1080/19490976.2023.2271629 (PMC10730206; doi:10.1080/19490976.2023.2271629)
Supplement: Supplemental Material [file KGMI_A_2271629_SM1253.docx]

**Estradiol mediates colonic epithelial protection in aged mice after stroke and is associated with shifts in the gut microbiome**

Supplementary material

**Supplementary Figure 1.**

**
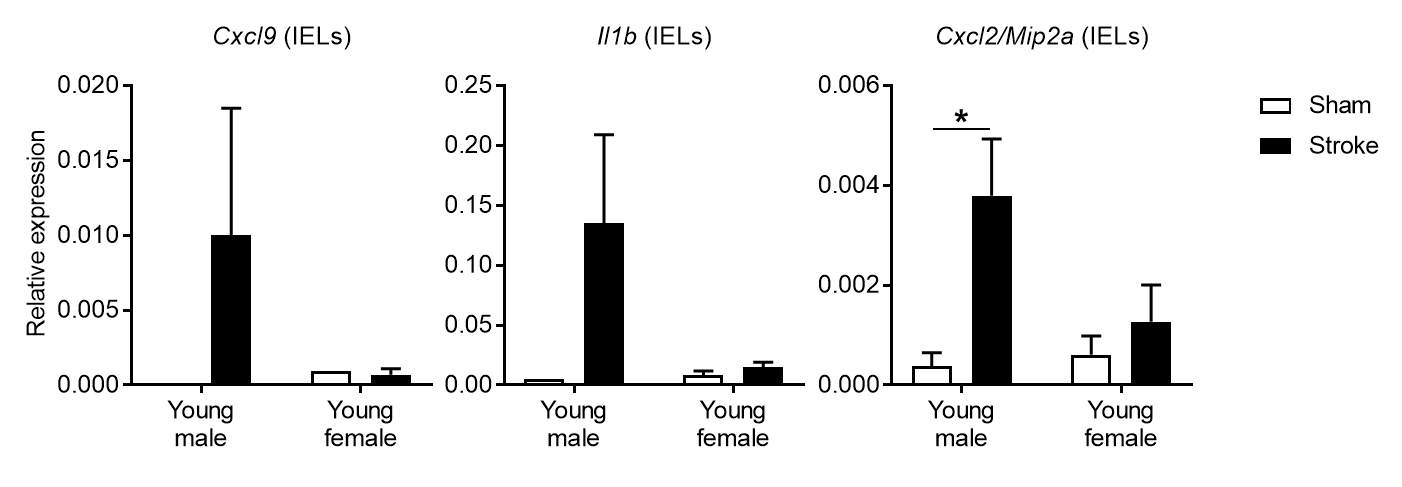
**

**Stroke increases proinflammatory genes in young male colonic IELs.**

At day 7 after MCAO, colonic intraepithelial lymphocytes (IELs) were isolated from mice using Percoll density gradients. Young male IELs exhibited increased expressions of proinflammatory genes including *Cxcl2* or *Mip2a*, not *Cxcl9* and *Il1b*. mRNA expression was examined using RT-qPCR. *Gapdh* was used to normalize mRNA levels of IELs. Data shown are mean±SEM. Two-way ANOVA method was used to analyze data with two factors including sex and control/stroke, followed by the post-hoc stroke vs control group comparison within male and female respectively. Multiple testing adjustment was performed by Sidak method. n=4 to 5 per group. *, *P*<0.05.

**Supplementary Figure 2.**

**
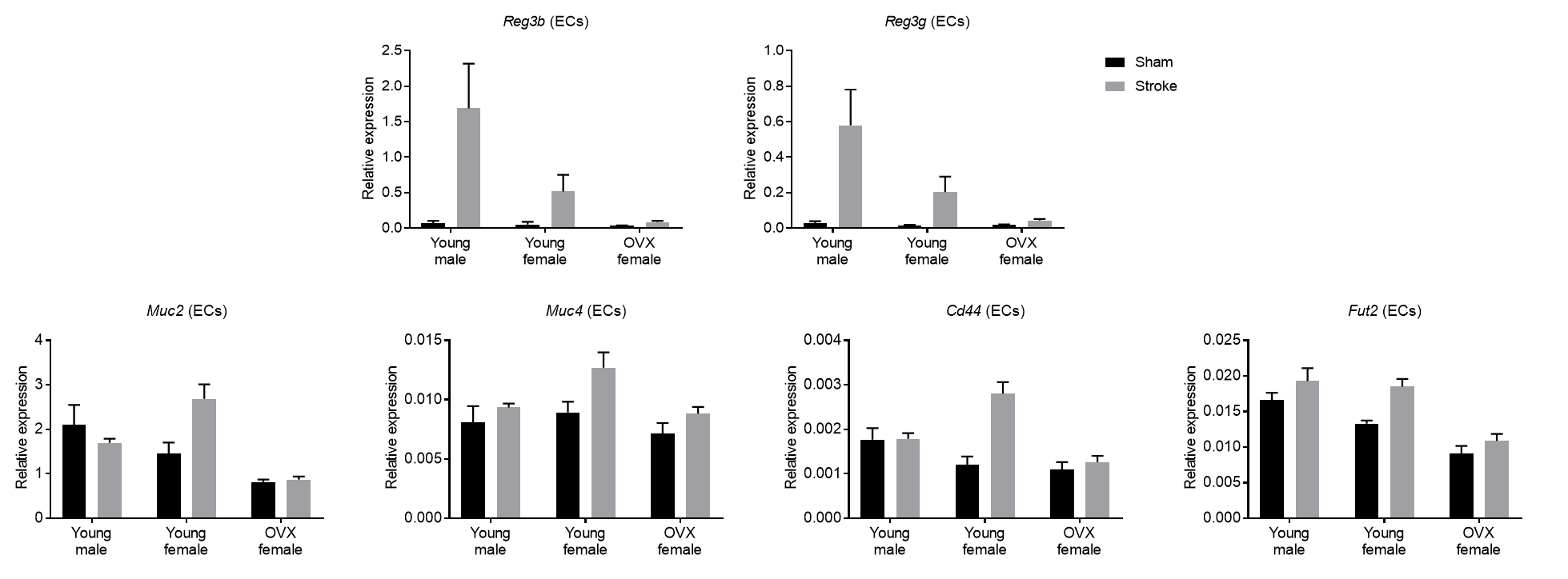
**

**Stroke-induced changes in gene expression in colonic epithelial cells (ECs) of young males and young females (both ovary-intact and ovariectomized (OVX) mice).** Figure 1b to d (males and females) and Figure 3b to d (females with and without OVX) were further directly compared.

**Supplementary Figure 3**.


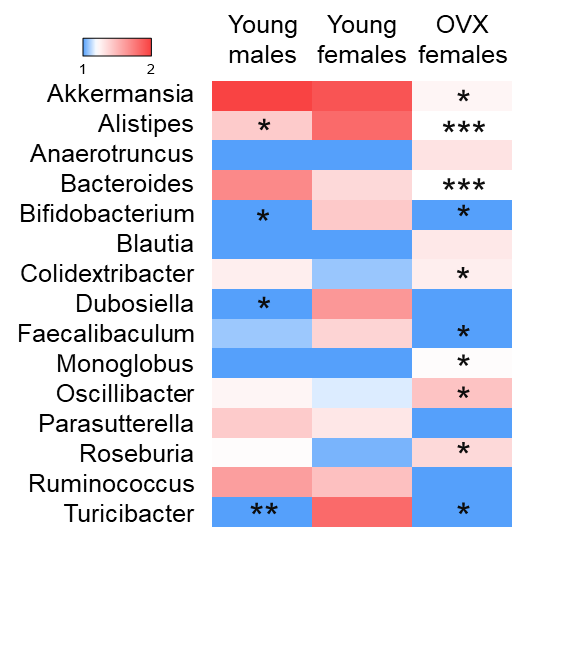


**Alterations in microbiota at genus level in young males and young ovariectomized (OVX) females compared to young ovary-intact females after stroke.** Heatmap illustrates relative abundance of the 15 most differentially expressed taxa at classified genus level between groups compared to young ovary-intact stroke females. Raw reads of identified genus are log 10-transformed to generate heatmaps to visualize directionality, each row graphically represents the median difference at post-stroke day 7 males and OVX females compared to ovary-intact stroke female group. n=3 to 5 per group. **P*<0.05, ***P*<0.01, ****P*<0.001; by unadjusted Mann-Whitney *U* tests comparing to ovary-intact stroke female group.

**Supplemental Figure 4**

**
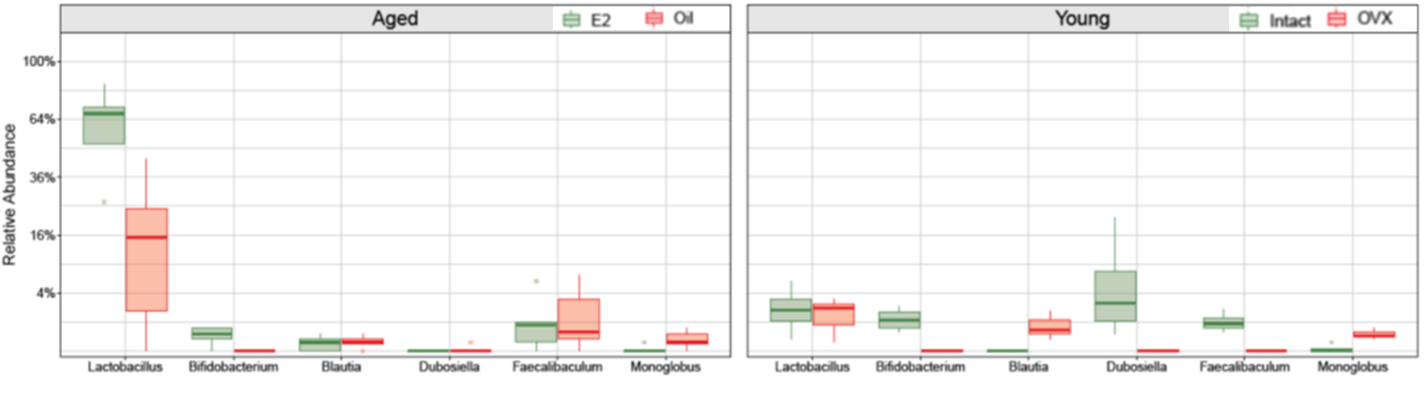
**

**Gut microbiota changes in young (intact and OVX) and aged (E_2_ and oil implanted) female mice after stroke.** Relative abundance comparisons revealed differences between groups.

**Supplementary Table 1. Primer sequences of the genes used in RT-qPCR.**

|  | Forward (5’ → 3’) | Reverse (5’ → 3’) |
| --- | --- | --- |
| *Actb* | GATCTGGCACCACACCTTCT | GGGGTGTTGAAGGTCTCAAA |
| *Cd44* | CCACAGCCTCCTTTCAATAACC | GGAGTCTTCGCTTGGGGTA |
| *Defa* | GGTGATCATCAGACCCCAGCATCAGT | AAGAGACTAAAACTGAGGAGCAGC |
| *Fut2* | TGTGACTTCCACCATCATCC | TCTGACAGGGTTTGGAGCTT |
| *Gapdh* | TGTGTGCGTCGTGGATCTGA | TTGCTGTTGAAGTCGCAGGAG |
| *Lyz1* | CTGTGGGATCAATTGCAGTG | CGGTTTTGACATTGTGTTCG |
| *Muc2* | CAAGTGATTGTGTTTCAGGCTC | TGGAGATGTTCTTGGTGCAG |
| *Muc4* | GACAAAGCACCAATTCCATCC | CCTTAGAGTTGCTGGTGATCTC |
| *Reg3b* | CTCTCCTGCCTGATGCTCTT | GTAGGAGCCATAAGCCTGGG |
| *Reg3g* | CCTGATGCTCCTTTCTCAGG | ATGTCCTGAGGGCCTCTTTT |
| *Reg4* | CTGGAATCCCAGGACAAAGAGTG | CTGGAGGCCTCCTCAATGTTTGC |
